# Microsecond hydrodynamic interactions in dense colloidal dispersions probed at the European XFEL

**DOI:** 10.1107/S2052252521006333

**Published:** 2021-07-28

**Authors:** Francesco Dallari, Avni Jain, Marcin Sikorski, Johannes Möller, Richard Bean, Ulrike Boesenberg, Lara Frenzel, Claudia Goy, Jörg Hallmann, Yoonhee Kim, Irina Lokteva, Verena Markmann, Grant Mills, Angel Rodriguez-Fernandez, Wojciech Roseker, Markus Scholz, Roman Shayduk, Patrik Vagovic, Michael Walther, Fabian Westermeier, Anders Madsen, Adrian P. Mancuso, Gerhard Grübel, Felix Lehmkühler

**Affiliations:** a Deutsches Elektronen-Synchrotron DESY, Notkestraße 85, 22607 Hamburg, Germany; b European X-ray Free-Electron Laser, 22869 Schenefeld, Germany; c The Hamburg Centre for Ultrafast Imaging, 22761 Hamburg, Germany; d Department of Chemistry and Physics, La Trobe Institute for Molecular Science, La Trobe University, Melbourne, VC 3086, Australia

**Keywords:** free-electron lasers, correlated fluctuations, dynamical studies, time-resolved studies, XFELs, nanoscience, SAXS, XPCS

## Abstract

Many soft-matter systems are composed of macromolecules or nanoparticles suspended in water. The characteristic times at intrinsic length scales of a few nanometres fall therefore in the microsecond and sub-microsecond time regimes. With the development of free-electron lasers (FELs) and fourth-generation synchrotron light-sources, time-resolved experiments in such time and length ranges will become routinely accessible in the near future. In the present work we report our findings on prototypical soft-matter systems, composed of charge-stabilized silica nanoparticles dispersed in water, with radii between 12 and 15 nm and volume fractions between 0.005 and 0.2. The sample dynamics were probed by means of X-ray photon correlation spectroscopy, employing the megahertz pulse repetition rate of the European XFEL and the Adaptive Gain Integrating Pixel Detector. We show that it is possible to correctly identify the dynamical properties that determine the diffusion constant, both for stationary samples and for systems driven by XFEL pulses. Remarkably, despite the high photon density the only observable induced effect is the heating of the scattering volume, meaning that all other X-ray induced effects do not influence the structure and the dynamics on the probed timescales. This work also illustrates the potential to control such induced heating and it can be predicted with thermodynamic models.

## Abstract 2

The dynamics of small nanoparticles dispersed in water are probed with multi-speckle XPCS taking advantage of the pulse structure of the European XFEL. The time resolution is sufficient to correctly probe the many-body hydrodynamic interactions in samples both at equilibrium and driven by the XFEL pulses. Practical examples demonstrate how X-ray pulse-induced dynamics can be controlled.

## 1. Introduction   

Colloidal suspensions, systems where particles of sizes in the nanometre to micrometre range are dispersed in a fluid, are one of the most studied classes of materials in modern condensed-matter physics. There are multiple reasons for this ranging from the fact that colloids can be used as a toy-model system able to describe more complex materials (Poon, 2015 ▸), to the possibility of fine-tuning single physical quantities (Israelachvili, 2011 ▸). Many macroscopic properties are determined by the dynamics of the particles in the liquid medium which, in turn, are heavily influenced by the particle concentration, quantified by the volume fraction Φ. The simplest case is found in diluted systems, where the particles only interact with the solvent and are therefore subjected to Brownian motion; conversely, in more concentrated systems the interactions between particles play an important role. The dynamics in concentrated suspensions of charge-stabilized nanoparticles are defined by a wide range of interactions, which differ both in nature and in effect. Already in a moderately crowded environment a particle is subjected to several long-range interactions, the most relevant being: (i) particle–particle electrostatic repulsion and (ii) solvent fluctuations that originate from movements of the other nanoparticles, known as hydrodynamic interactions (HIs) (Beenakker & Mazur, 1983 ▸). The latter is a many-body effect, which presents challenges in its analytic and computational description. The effects of HIs are more evident in the intermediate scattering function *f*_*q*_(*t*), which is the *q*-component of the normalized number-density correlation function (Hansen & McDonald, 2013 ▸). HIs have been measured by dynamic light scattering (Pusey & Tough, 1983 ▸; Pusey & van Megen, 1983 ▸) and are nowadays typically probed with X-ray photon correlation spectroscopy (XPCS) experiments, a well established technique in the study of dynamical processes on the nanometre and sub-nanomatre length scales (Sutton *et al.*, 1991 ▸; Abernathy *et al.*, 1998 ▸; Grübel *et al.*, 2008 ▸; Shpyrko, 2014 ▸; Madsen *et al.*, 2016 ▸; Sandy *et al.*, 2018 ▸; Lehmkühler *et al.*, 2021 ▸). For diffusing systems the intermediate scattering function can be described by 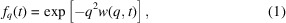
where *w*(*q*, *t*) is the associated collective width function (Banchio *et al.*, 2018 ▸; Martinez *et al.*, 2011 ▸). It is then possible to identify a timescale 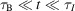 in which *f*_*q*_(*t*) follows a simple exponential relaxation, where 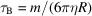 is the momentum relaxation and 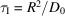 is the interaction time (Nägele, 1996 ▸); where *m* is the mass of a single nanoparticle, η is the solvent viscosity and *R* is the particle radius. In this case 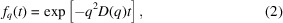
with 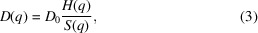
where 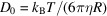 is the translational diffusion constant given by the Stokes–Einstein equation, *S*(*q*) is the static structure factor and *H*(*q*) is the function that quantifies the HI. Experimentally it is possible to access the *q*-dependent diffusion constant by looking at the short time limit of *w*(*q*, *t*), which, in practice, means (Martinez *et al.*, 2011 ▸; Westermeier *et al.*, 2012 ▸)
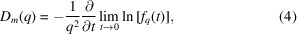
where the limit 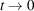 is justified by the fact that τ_B_ is typically in the picosecond time regime, which is several orders of magnitude faster than the time resolution of typical photon correlation experiments. The particles in colloidal suspensions are often found with a distribution of sizes (dispersity), which has implications on both dynamical and structural properties of the system. Neglecting the charge distribution, it is possible to describe the experimentally accessible structure factor [*S_m_* (*q*)] in terms of the ‘ideal’ monodisperse *S*(*q*) and the decoupling amplitude factor *X*(*q*) (Nägele, 1996 ▸; Westermeier *et al.*, 2012 ▸) via 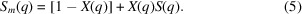
The size dispersity affects dynamical properties in a similar way. Under the same decoupling approximation employed for *S*(*q*), we can modify Equation (3) to 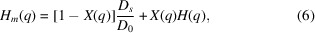
where *D*_*s*_ is the short-time translational self-diffusion coefficient of a representative particle in a quiescent suspension of directly and hydrodynamically interacting particles (Westermeier *et al.*, 2012 ▸). It is then possible to analytically express an approximation of *H*(*q*) taking the structure factor *S*(*q*) as the only input parameter with the lowest-order δγ-expansion results by Beenakker and coworkers (Beenakker & Mazur, 1983 ▸, 1984 ▸; Beenakker, 1984 ▸; Heinen *et al.*, 2011*a* ▸).

Similarly to the structure factor, *H*(*q*) has the highest impact in the *q*-ranges corresponding to the first-neighbour shells and the magnitude of the diffusion constants remains similar to *D*_0_, unless the volume fraction is too high and the system approaches a glassy state. Consequently, a time resolution of microseconds is required to probe the dynamics of colloidal particles dispersed in water by XPCS or the related technique X-ray speckle visibility spectroscopy (XSVS). The main obstacles are represented by the short exposure times (Möller *et al.*, 2019 ▸) and the low count rates (Jo *et al.*, 2021 ▸). This challenges the measurements of faster timescales even for simple systems and dedicated detectors (Zhang *et al.*, 2018 ▸) and complicates its application for more delicate samples (Vodnala *et al.*, 2018 ▸; Lurio *et al.*, 2021 ▸). For these reasons, previous experiments that aimed to measure *H*(*q*) usually had to rely on combinations of point-detectors and the addition of viscous solvents to slow down the dynamics into the millisecond regime (Riese *et al.*, 2000 ▸; Robert *et al.*, 2008 ▸). However, with the development of the European X-ray Free Electron Laser (European XFEL), sequential XPCS experiments on the sub-microsecond timescale have become possible, allowing the observation of diluted colloidal systems both at (quasi-) equilibrium and under a driven condition (Lehmkühler *et al.*, 2020 ▸). The question arises of how the XFEL beam affects more complex systems. Hence, in the present work we will extend the analysis to more concentrated systems where inter-particle interactions and HIs play a crucial role in the internal dynamics. Many nanoparticles, macromolecules and proteins are found in water which is their native environment, and microsecond-XPCS studies are an important tool for solving many questions in fields ranging from soft-matter physics and biology to nanoscience. Our findings, on a prototypical soft-matter system, will be helpful in the design and interpretation of future XFEL experiments.

## 2. Materials and methods   

**Figure 1:**
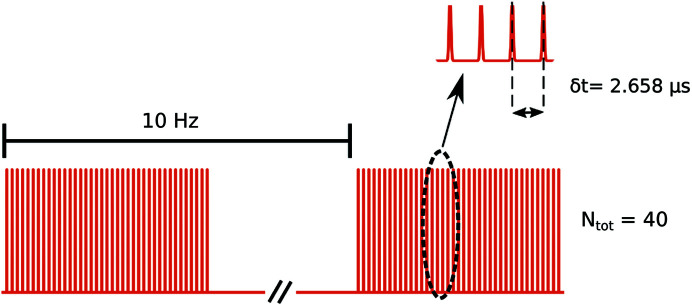
Schematic of a possible pulse scheme. The trains were composed of *N*_tot_ = 40 pulses separated by δt = 2.658 µs; each train is produced with a frequency of 10 Hz. The dashed ellipse highlights a set of pulses reported above on a magnified *x*-scale. The horizontal axis is not to scale.

The samples were made starting from commercially available strongly screened charge-stabilized silica nanoparticles (Sigma–Aldrich, Ludox TMA 420859). The charge stabilization can be attributed to surface charging of the silica nanoparticles in aqueous solvents, and the screening is carried out by the supplier with the addition of salts. The product is typically sold in large volumes at a nominal mass concentration of 34 wt%. While the mass concentration between different batches is typically consistent, the size and dispersity of the nanoparticles can vary significantly. For the main part of this experiment, three different concentrations were produced by adding ultra-pure water to one original dispersion (sample A) to reach concentrations of one half (sample B) and one fourth (sample C) of the initial solution. The samples were placed in thin-walled quartz capillaries (diameter 0.7 mm, wall thickness 10 µm), sealed with hot glue and placed in the experimental chamber with a custom-made sample holder. The experiment was performed at the SPB/SFX instrument, please refer to the work by Mancuso *et al.* (2019 ▸) for a more detailed description. The photon energy was set at 9.3 keV with an average pulse energy per run (thus averaged over train and pulses) of 1.7 mJ with a relative standard deviation of 0.09. Intensity fluctuations are a consequence of the self amplified spontaneous emission (SASE) process at the basis of modern XFELs. The X-ray flux delivered at the sample position was controlled with stacks of silicon attenuators in the beam path with total thicknesses ranging from 1.2 to 0.6 mm, taking into account the transmission of the beamline optics, the incoming beam was reduced by a factor between 1.8 × 10^−6^ and 7 × 10^−4^. The measurements were repeated at least twice for each attenuator value. The pulses were focused on the samples by a KB mirror system (Bean *et al.*, 2016 ▸) to a focal spot size of about 4.4 × 3.6 µm (H × V). The speckle patterns were measured 5.5 m downstream of the sample with the 1M pixel Adaptive Gain Integrating Pixel Detector (AGIPD) (Allahgholi *et al.*, 2019*a* ▸,*b* ▸). The European XFEL has a particular pulse structure, in which bunches of pulses (trains) with repetition rates in the megahertz range are sent to a given experiment every 0.1 s (10 Hz), an example of a possible pulse scheme is depicted in Fig. 1 ▸. In the present experiment each sample was measured with runs of 500 trains, and three different pulse schemes were employed. A single train consisted of series of *N*_tot_ pulses separated by a certain time delay δ*t*. Unless stated otherwise, the configuration with *N*_tot_ = 120 and δ*t* = 886 ns (1.128 MHz repetition rate) was used; some samples were also probed with *N*_tot_ = 60, δ*t* = 1.772 µs and *N*_tot_ = 40, δ*t* = 2.658 µs. The samples were moved vertically during the acquisition in order to expose a fresh spot for every train. The raw data from the AGIPD were then elaborated by custom-made software; for more details see the supporting information or the work by Lehmkühler *et al.* (2020 ▸). At a later stage, additional data were taken at the MID instrument [see the work by Madsen *et al.* (2021 ▸) for further information] specifically designed for coherence applications. The parameters were 9 keV photon energy, 7.3 m sample-to-detector distance and ∼10 µm × 10 µm beam size, and repetition rates of 2.256, 1.128 and 0.564 MHz. The detector was also a 1M pixel AGIPD and the XFEL pulse energies per pulse ranged in the same interval of the SPB/SFX experiment; however, the sample was from a different batch and was measured only at the nominal concentration of 34 wt%. The MID instrument controls the flux with stacks of CVD diamond windows, which for the data reported here had total thicknesses ranging from 2.5 to 4.5 mm. Moreover, the flux was additionally reduced by a pin-hole before the focusing optics and an air path before the sample. The intensity was then reduced by a factor in the range 1.4 × 10^−4^ to 1.5 × 10^−3^. XPCS correlates pairs of speckle patterns sampled at two times *t*_1_ and *t*_2_ = *t*_1_ + *t* producing a two-times correlation matrix, see for example (Malik *et al.*, 1998 ▸; Sutton *et al.*, 2003 ▸; Duri *et al.*, 2005 ▸): 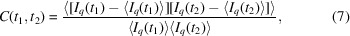
with the ensemble average 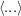 performed over all the pixels belonging to the same *q*-value. *C*(*t*_1_, *t*_1_ + *t*) holds important information when the investigated dynamics are not ergodic, enabling additional insight into out-of equilibrium conditions (Bikondoa, 2017 ▸; Madsen *et al.*, 2010 ▸). However, if the dynamics are stationary the correlations will depend solely on the lag time *t* = *t*_2_ − *t*_1_, and with an additional time average of the two-time matrix it is possible to extract the correlation function *g*_2_(*t*) −1. The latter can be linked to the ISF via the Siegert relation: 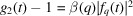, where β(*q*) is the speckle contrast determined by the experimental conditions (Sutton, 2008 ▸). In the present experiment β(*q*) ranged between 0.21 and 0.3 for the probed *q* regions at SPB/SFX. The characterization was performed with static samples and is reported in the work by Lehmkühler *et al.* (2020 ▸). Due to the different experimental conditions, such as beam size and sample-detector distance, the contrast in the MID experiment ranged between 0.08 and 0.12 in the *q* region reported here. In a diffusing system 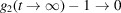, but some static contributions such as the *I*(*q*) or some ‘unevenness’ in the detector chips will lead to small deviations from the ideal limit (Duri *et al.*, 2005 ▸), resulting in a baseline that originates from purely static and instrumental contributions. In the present work, *g*_2_(*t*) − 1 was computed for each train of pulses. After filtering away the data produced by weak trains, all the correlations calculated for a given *q* were averaged together. Lastly, the averaged *g*_2_(*t*) − 1 were fitted with a stretched exponential function: 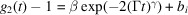, where Γ is the relaxation rate and 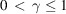. This step allowed us to ascertain the value of the ‘instrumental’ baseline (*b_i_*) and correct the correlations in order to obtain the right value for 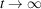. The presence of this instrumental baseline, which could reach up to 12% of the contrast in the probed *q*-region, was also observed in data obtained from diluted nanoparticles measured within the same experiment and documented by Lehmkühler *et al.* (2020 ▸). The exact values of *b_i_* depend on many factors, such as the status of the AGIPD or the energy stability within a train, therefore the correction must be done at least for every run. The values obtained in the diluted samples from Lehmkühler *et al.* (2020 ▸) and the concentrated ones of the present paper are similar. Moreover, in the data from MID, which are obtained with an upgraded version of the raw data correction pipeline, the values of *b_i_* are typically lower than 0.2% of the corresponding contrast. For these reasons we can conclude that, despite the relatively high volume fractions, the samples are decorrelated completely on the probed timescale.

## 3. Results   

### 3.1. Structure   

**Figure 2:**
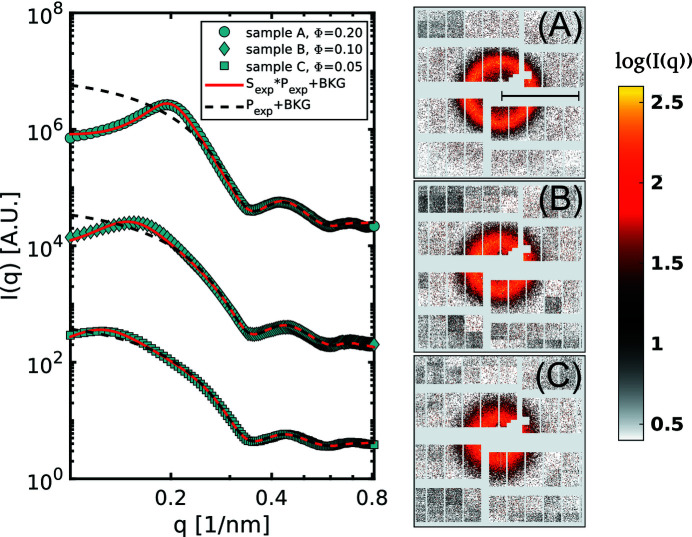
Structural information for the three samples A, B and C. Cyan points: scattered intensity *I*_exp_(*q*), black dashed lines: form factor, red lines: *I*(*q*) fitted with Equation (8). For clarity, the curves are multiplied by a factor. On the right-hand side, single-shot speckle patterns from the three samples are reported. In order to highlight the speckles, a median filter has been applied to the images. The AGIPD is not optimized for SAXS-XPCS experiments, due to the presence of large gaps between the modules and the necessity to mask large areas of the detector. Nevertheless, it is still possible to obtain high-quality data owing to the high repetition rate of this device. The black bar in the pattern (A) indicates a distance of 0.5 nm^−1^ in the scattering plane.

The AGIPD is capable of recording a speckle pattern from every pulse resulting in about 60 000 frames for each run. Every single frame was then azimuthally averaged in order to obtain the *I*(*q*). Once we verified that XFEL pulses do not affect the structure of the sample during the measurement, indicated by the absence of any systematic trends in the *I*(*q*) as a function of train or pulse number (see the supporting information for further details), all the individual *I*(*q*) were averaged to obtain the curves shown in Fig. 2 ▸. The data were then fitted with the function: 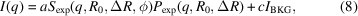
where *I*_BKG_ is the background intensity measured from capillaries filled with pure water, and *a* and *c* are simple scaling factors. The experimental form factor 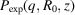 takes into account the finite resolution of the detector (see the supporting information). The dispersity of the nanoparticles is modelled with a Schulz–Zimm distribution (Zimm, 1948 ▸; Schulz, 1982 ▸). From each fit we obtained three independent measurements of the mean radius, all compatible with each other, which gives us *R*_0_ = 12.6 ± 0.1 nm and a size dispersity of Δ*R*/*R*_0_ = 0.11 ± 0.02. The experimental structure factor *S*_exp_ can be simplified with the decoupling approximation of Equation (5) in the two terms *X*(*q*) and *S*(*q*). Under the assumption that the direct particle interaction is described by a Derjaguin–Landau–Verwey–Overbeek (DLVO) pair potential (Marshall, 1949 ▸), it is possible to analytically express *S*(*q*) with the MPB-RMSA method described by Heinen *et al.* (2011*b* ▸). From the fit of the *S*(*q*) we were also able to obtain measurements of the actual volume fractions which are Φ_A_ = 0.20, Φ_B_ = 0.1 and Φ_C_ = 0.05 for samples A, B and C, respectively, which are in good agreement with the predicted values.

### 3.2. Dynamics   

**Figure 3:**
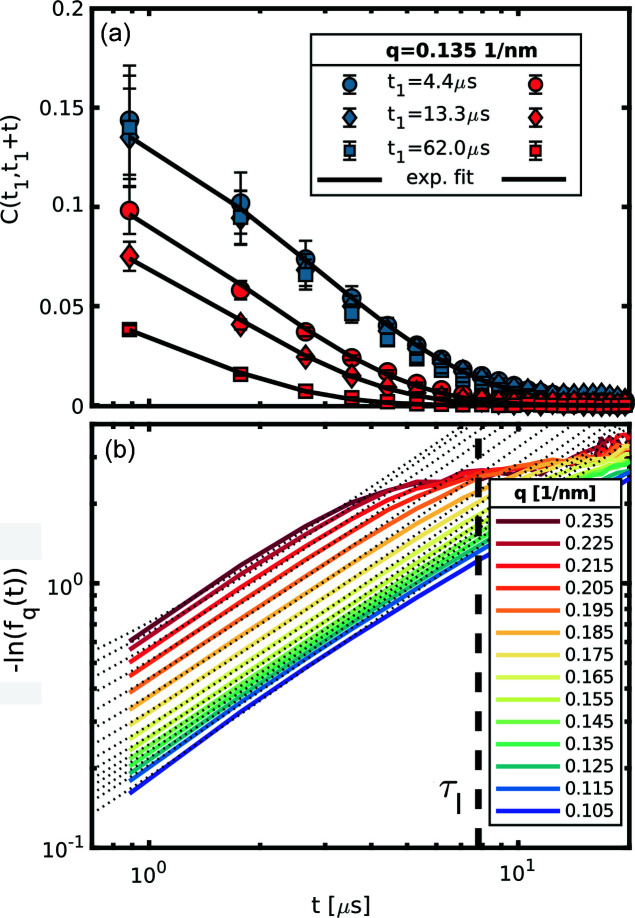
(*a*) Correlations obtained after 4.4 µs (circles), 13.3 µs (diamonds) and 62.0 µs (squares) hitting the sample with a fluence of 3.9 mJ mm^−2^ (blue symbols) and 10.5 mJ mm^−2^ (red symbols). The black lines are exponential fits to the data. Within the sensitivity given by the XFEL intensity fluctuations, the dynamics within a train of pulses remain stationary for the lower fluence, whereas in the higher fluence the relaxations are faster for larger *t*_1_. For clarity only the correlations from one *q* of sample B are reported, but other *q*s in other samples provide the same qualitative information. The error bars on the data points are obtained from the variance of the two-time correlation matrix. (*b*) 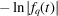 for sample B. All the ISFs are completely decorrelated (*i.e.* the measurement noise overcomes the dynamic signal) after reaching the interaction time τ_*I*_, here highlighted by the black dashed line. It is evident that, for higher *q*, the useful portion of the ISF is rather limited. The dotted lines are the fits to Equation (9) over the appropriate time regime.

**Figure 4:**
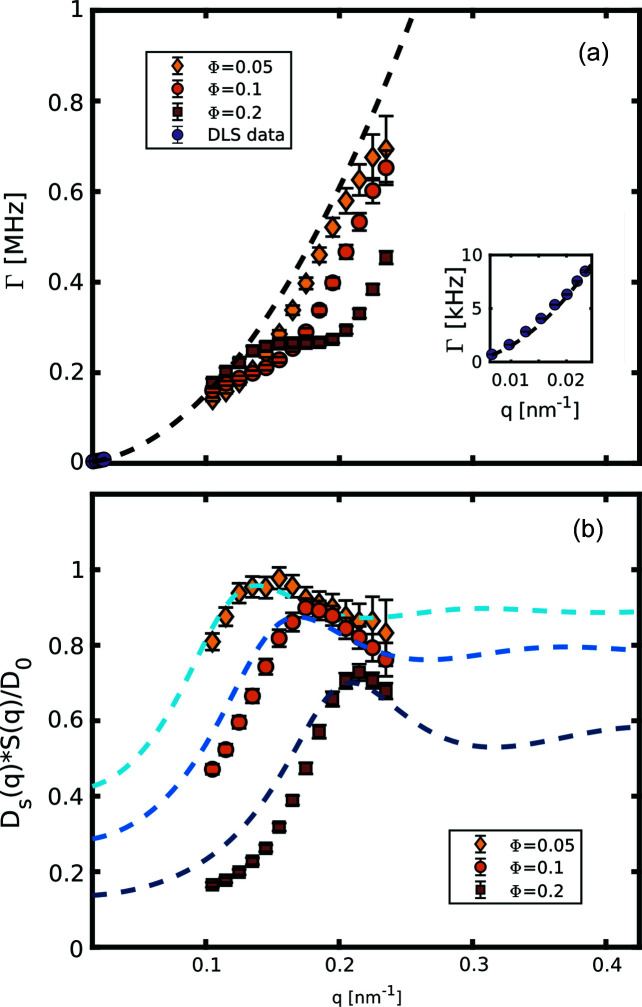
(*a*) Relaxation rates as a function of temperature for sample A (red squares), sample B (orange circles) and sample C (yellow diamond). The data are reported together with the pure diffusive behaviour (dashed line) extrapolated from DLS measurements on a diluted sample at 30°C (purple circles). The detail of the DLS data is reported in the inset. (*b*) Experimental HI for samples A, B and C as a function of *q*. The data points are reported with the respective *H_m_* (*q*) obtained from the analytical approximations reported by Westermeier *et al.* (2012 ▸) and Heinen *et al.* (2011*a* ▸).

The pulses within the same train show a remarkable stability, quantified by the analysis of the speckle contrast reported by Lehmkühler *et al.* (2020 ▸). This property, together with the high repetition rate, are key aspects that enable the sequential XPCS analysis on microsecond timescales. From each train the two-time correlation matrix 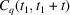 is obtained, and from that, the *g*_2_(*t*) is derived. All these correlation functions were then averaged in order to obtain the intermediate scattering function for different *q* values. The measurements are repeated for several intensities of the XFEL pulses, in order to quantify the effects of the XFEL pulses on the sample. Fig. 3 ▸(*a*) shows some elements of 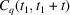 for different values of *t*_1_ obtained at two different fluences (here and in the rest of the paper the fluence values are given per pulse). The dynamics remains effectively stationary within a train for fluences up to 3.9 mJ mm^−2^, while for fluences of 10.5 mJ mm^−2^ there is a marked dependence on *t*_1_ in accordance with Lehmkühler *et al.* (2020 ▸). Equation (2) implies that we can approximate 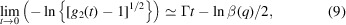
where 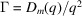. Thus, the short time diffusion can be obtained with a simple linear regression of the initial part of the ISF keeping in mind that the interaction time for our system is τ_*I*_ ≃ 7.8 µs. In Fig. 3 ▸(*b*) an example of the linearized ISF from sample B at 3.9 mJ µm^−2^ is reported (coloured lines) together with their respective linear fits (dotted lines). In this case, the detachment from the linear trend in the experimental data is mainly due to the decreasing signal to noise ratio for larger lag times, as also shown by Banchio *et al.* (2018 ▸). Due to the relatively narrow dynamical range of the present experiment, the correlations at larger *q* are sampled only in the last part of their relaxation curve. In this situation the assumptions of Equation (9) no longer hold, hence the diffusion constant for the faster ISFs is obtained with a simple exponential fit of the *g*_2_(*t*) functions fixing the contrast to the value observed in static reference samples, similarly to what has been performed by Lehmkühler *et al.* (2020 ▸). In Fig 4 ▸(*a*), the relaxation rates Γ as a function of *q* are reported; as a comparison the purely diffusive behaviour is also plotted, obtained with the extrapolation of dynamic light scattering (DLS) measurements performed on diluted suspensions. These latter measurements were performed with the commercial apparatus LS Spectrometer from LS Instruments. In all three samples we can observe the typical de Gennes narrowing effect (De Gennes, 1959 ▸), manifested as a deviation from the simple *q* ^−2^ law, with a dynamical slowing that becomes more pronounced in correspondence of their respective structure factor peaks. Inverting Equation (8) we can obtain a good estimate of the real structure factor *S_m_* (*q*). With this information the experimental 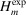 can be obtained via 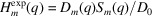, reported in Fig. 4 ▸(*b*) together with computed 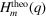 from Equation (6) (dashed lines). Both 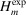 and 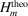 maintain a value below 1 in the probed *q*-range. Thus the HIs act as a frictional force slowing down the dynamics. Moreover, the fact that even at volume fractions of 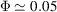 the peak value of the hydrodynamic function is still below 1 suggests that the inter-particle potentials are heavily screened and the system is in a situation close to the hard-sphere limit (Westermeier *et al.*, 2012 ▸). Moreover, the height and width of the first peak of *H_m_* (*q*) decrease with increasing volume fractions, in agreement to what has been observed in hard-sphere systems both theoretically (Banchio & Nägele, 2008 ▸) and experimentally (Orsi *et al.*, 2012 ▸). The approximated models for 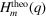, computed with the code developed by Westermeier *et al.* (2012 ▸), are able to qualitatively reproduce the data, but fail progressively for smaller *q* and higher concentrations. This discrepancy can arise for several reasons: (i) despite the fact that the XFEL repetition rate allows a clear measurement of the ISF relaxation, the short time limit is still not properly sampled; (ii) the Ludox is not a system composed of pure nanoparticles dispersed in water, because a non-negligible number of stabilizing ions are present and we are approaching the limits of the approximations of the δγ-expansion (Heinen *et al.*, 2011*a* ▸) at the basis of our model.

### 3.3. High-fluence data   

**Figure 5:**
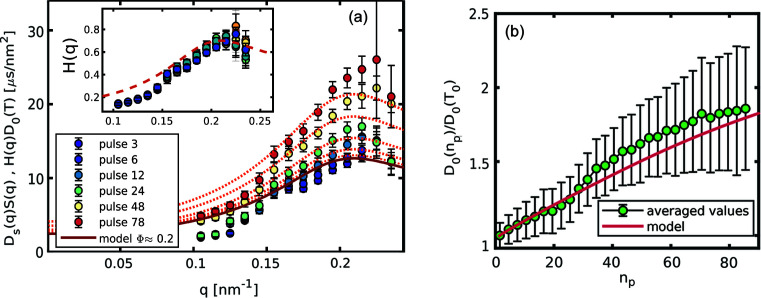
Dynamical quantities as a function of the number of pulses for sample A exposed to 10.5 mJ mm^−2^. (*a*) Contribution of the HIs to the diffusion constant for various *n_p_*. The lines are the equilibrium *H_m_* (*q*) multiplied by the expected diffusion constant. In the inset the data points are rescaled by the expected diffusion constant and plotted together with the equilibrium *H_m_* (*q*). (*b*) Relative change in the diffusion constant; the values are obtained from the average over *q* of 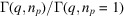, and the error bars from the propagation of the uncertainty of the fitted relaxation rates, expressing a systematic error originating from a reduced time resolution at larger *n_p_*. The red line is the change in *D*_0_(*T*) as expected from the time-resolved model by Lehmkühler *et al.* (2020 ▸).

As pointed out by Lehmkühler *et al.* (2020 ▸) and Hruszkewycz *et al.* (2012 ▸), the high brilliance of the XFEL pulses can strongly affect the probed samples without necessarily causing permanent damage to them. Each pulse deposits a considerable amount of energy which, in absence of chemical reactions, is converted into heat. The scattering volume finds itself at higher temperatures than the surrounding sample, and will try to relax back to the initial condition. However, the high repetition rate of the XFEL prevents a complete temperature equalization and will drive the system into a heated state. This introduces an explicit dependence of the dynamics on the number of pulses that reach the sample (*n_p_*) on all the observed dynamical quantities. Additionally, colloids are heterogeneous systems and the nanoparticles, in general, will absorb a different amount of energy with respect to the solvent. The dynamics of the colloids will correspond then to an effective temperature which differs substantially from the average temperature of the scattering volume (Rings *et al.*, 2010 ▸; Lehmkühler *et al.*, 2020 ▸). However, in the present case the nanoparticles are fairly small, meaning that the time required to reach the temperature of the surrounding water is ∼62 ns (see the supporting information), and the high concentrations introduce a small but not negligible amount of deposited heat in the probed sample. Therefore, within the microsecond time resolution of this experiment, the whole scattering volume can be considered at the same temperature. Similarly to that discussed in the previous section, a time-dependent hydrodynamic interaction can be extracted from the product 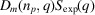. As shown in Fig. 5 ▸(*a*) the general features of *H*_exp_(*q*) are conserved for all values of *n_p_*. If we normalize the 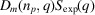 by the time-dependent diffusion constant *D*_0_(*n_p_*), all data points collapse on the equilibrium *H*(*q*) as illustrated in the inset. This suggests that the only effect is due to the changing temperature in the scattering volume. Thus, the HIs are not affected by the out-of-equilibrium condition of the heated sample for the investigated fluence and timescale ranges. This can be expected since *H*(*q*) is the result of all the movements of the nanoparticles mediated by the solvent, hence the fastest movements of a given particle will be transmitted at the same velocity of the sound waves in the liquid medium (Henderson *et al.*, 2002 ▸). Movements in the micrometre to nanometre length-scale will propagate in the nanosecond to picosecond time regime, which is still orders of magnitude faster than the other processes probed in this experiment. This indicates that the only quantities affected by the XFEL pulses are the explicitly temperature-dependent ones, *i.e.* *D*_0_ and η(*T*). In Fig. 5 ▸(*b*) the behaviour of 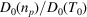 is reported. The evolution of the temperature-dependent diffusion constant displays a simple monotonic growth. The time-resolved model introduced by Lehmkühler *et al.* (2020 ▸) provides an appropriate description of the observed dynamics even in this case [red line of Fig. 5 ▸(*b*)] giving us also the possibility to model the time evolution of the effective temperature in our scattering volume. The only way to outrun this heating effect is to probe on faster timescales, *e.g.* in the picosecond range with different techniques using split and delay devices as described elsewhere (Roseker *et al.*, 2018 ▸, 2020 ▸; Hirano *et al.*, 2018 ▸; Sun *et al.*, 2019 ▸). However, with such short time intervals one crosses the limit represented by the momentum relaxation τ_B_, and the description of Equation (2) is no longer valid.

### 3.4. Control of the heating effect   

**Figure 6:**
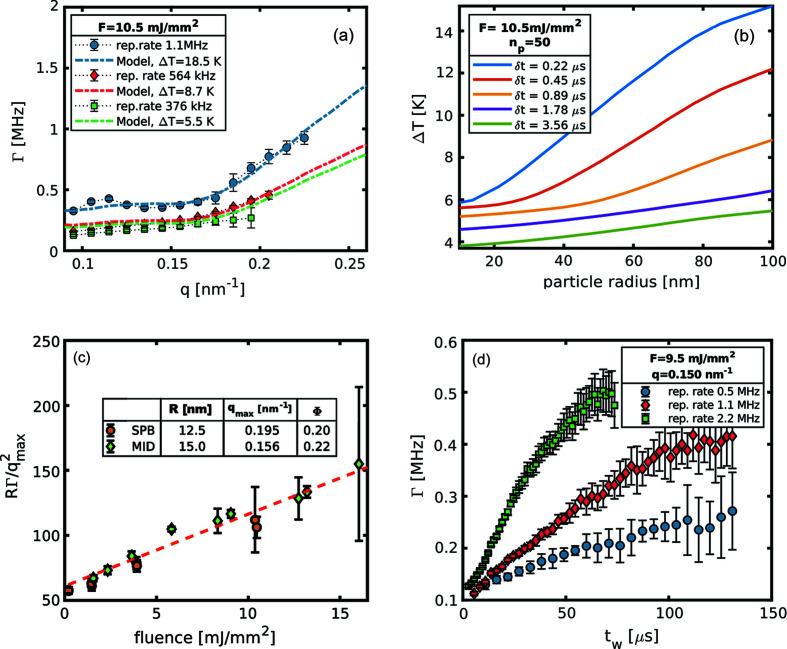
Possible strategies to control the beam-heating effect. (*a*) Relaxation rates from sample B are shown in three different configurations as indicated, the respective dashed lines are the average relaxation rate obtained from the time-resolved model. (*b*) Expected average temperature jump in a diluted colloidal sample of silica nanoparticles as a function of the particle radius for different repetition rates at a fluence of 10.5 mJ mm^−2^ and 50 pulses. (*c*) Rescaled relaxation rates after 15 pulses as a function of the fluence for the SPB/SFX and MID data. Once the differences due to the different particle size are removed, the diffusion constant follows the same linear behaviour for both systems, confirming the fact that the only changing quantity is the temperature in the scattering volume. (*d*) Γ(q*) for different waiting times at three different repetition rates measured at MID.

Depending on the kind of experiment performed, beam-induced heating can be an interesting tool to exploit or an additional complication to be taken care of. The only way to mitigate this effect, without renouncing to the XFEL’s high number of photons per pulse, is to reduce the energy density deposited in the scattering volume. This can be achieved either by modifying the single-pulse fluence, *e.g.* using larger beam sizes, or by changing the number of pulses and the repetition rate of the pulses. An example of this latter approach is shown in Fig.6 ▸(*a*), where both the repetition rate and number of pulses were modified in order to sample the same time window. One can observe how reducing the deposited energy by a factor of 3 and giving more time to dissipate the heat reduces the increase of the sample temperature. In the present case, reducing the repetition rate translates to an almost linear reduction of the observed temperature owing to the small size of the particles. Conversely, for larger nanoparticles which require more time to equilibrate their temperature with that of the bulk solvent, the effective temperature starts to build up as the repetition rate of the XFEL approaches this characteristic time [see Fig.6 ▸(*b*)]. Aside from the repetition rate, the other key quantity that controls the heating of a sample is the fluence per pulse, and this can be seen by comparing the results obtained in two different experiments at two different instruments (SPB/SFX and MID). However, we have to make some preliminary considerations. In fact, the measurements carried out at MID were performed on a different batch of nanoparticles, with the consequence that size (*R* = 15.0 ± 0.1 nm) and concentration (Φ = 0.22) are slightly different from those of the sample described in the previous sections. Nevertheless, it is still possible to compare the results of the two experiments. Considering two suspensions with different particle sizes (*R^a^* and *R^b^*), the translational diffusion constants will differ by the ratio between the two radii, hence 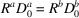. Furthermore it can be shown (Banchio & Nägele, 2008 ▸) that, at the peak of the structure factor, both *S*(*q*) and *H*(*q*) depend solely on the volume fraction. Thus, for similar concentrations one can write 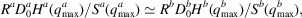
where 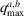 indicates the peak position of the structure factor. In Fig. 6 ▸(*c*) we can see the rescaled diffusion constants for the two batches measured after 15 pulses at different fluences. Despite being obtained from samples with different particle sizes, the data from the two experiments agree quite well, scaling the same way with the incoming flux. As expected, all the data follow a linear behaviour due to the fact that the rising temperature of the water in the scattering volume is proportional to the fluence, see the supporting information or the work by Lehmkühler *et al.* (2020 ▸). For fluences lower than 2.5 mJ mm^−2^ the relaxation rates become less sensitive to the incoming flux. Eventually for even lower fluences, not probed within the present work, the diffusion constant will reach the stable value of a completely unperturbed system. However, the exact value of this threshold depends strongly on the details of the samples. For example, in a system of larger particles but much more diluted, reported by Lehmkühler *et al.* (2020 ▸), it is possible to see that fluences up to 3.9 mJ mm^−2^ do not affect the dynamics, but past that threshold the heating effects become much more severe as illustrated in Fig.6 ▸(*b*). In Fig. 6 ▸(*d*) Γ(*q*_max_) is shown as a function of the time elapsed since the first pulse reached the sample. In this situation a lower repetition rate means both more time for the thermal relaxation and fewer pulses on the sample producing a milder heating of the scattering volume. Lastly, reducing the repetition rate (or in general the number of pulses) has the drawback of a limited time window that can be probed in a correlation experiment.

## 4. Conclusions   

We have reported a sequential microsecond XPCS study at the European XFEL on colloidal systems of charge-stabilized nanoparticles at different concentrations, both in stationary and driven conditions. In low-fluence regimes it is possible to maintain the scattering volume in equilibrium with a temperature only slightly higher than the thermal bath. In all three samples measured in such a regime, the observed dynamics are heavily influenced by particle–particle interactions and HIs. Moreover, the *q*-dependence of the diffusion constant can be well described theoretically. This indicates that it is possible to sample equilibrium dynamics with a sub-microsecond repetition rate XFEL. For higher X-ray fluencies, the system is continuously driven towards higher temperatures, in accordance with the observations of Lehmkühler *et al.* (2020 ▸) for diluted suspensions. The temperature increase, happening on the microsecond timescale, can still be described with a classical approach. Most importantly, it does not modify the HIs, which can still be considered almost instantaneous in the investigated time regime. We also illustrated some strategies that can be helpful for mitigation or control of the X-ray beam-heating effect. Lastly, in all the investigated X-ray intensity regimes, no noticeable changes in the structure factor have been observed, implying that no substantial changes in the inter-particle potentials happen as a consequence of the XFEL illumination. This means that only temperature effects are present and other effects, *e.g.* change of charge, creation of radicals *etc.* cannot disturb the system on the probed length- and timescales. This study is another confirmation that the paradigm ‘measurement before destruction’ of the first XFELs has been overcome at modern-day facilities. The nanoparticles observed here are known to be quite robust against hard X-rays; for more delicate samples (*e.g.* biomolecules) the limitations will surely be more severe but there is no indication that equilibrium dynamics should not be accessible. 

## Supplementary Material

Details for better understanding data analysis and theoretical models. DOI: 10.1107/S2052252521006333/it5024sup1.pdf
