# Supplementary material for: Microsecond hydrodynamic interactions in dense colloidal dispersions probed at the European XFEL
Source: IUCrJ. 2021 Jul 28;8(Pt 5):775–83. doi: 10.1107/S2052252521006333 (PMC8420773; doi:10.1107/S2052252521006333)
Supplement: Supplementary file 1 [file m-08-00775-sup1.pdf]

IUCrJ

Volume 8 (2021)

Supporting information for article:

Microsecond hydrodynamic interactions in dense colloidal dispersions probed at the European XFEL

Francesco Dallari, Avni Jain, Marcin Sikorski, Johannes Möller, Richard Bean, Ulrike Boesenberg, Lara Frenzel, Claudia Goy, Jörg Hallmann, Yoonhee Kim, Irina Lokteva, Verena Markmann, Grant Mills, Angel Rodriguez-Fernandez, Wojciech Roseker, Markus Scholz, Roman Shayduk, Patrik Vagovic, Michael Walther, Fabian Westermeier, Anders Madsen, Adrian P. Mancuso, Gerhard Grübel and Felix Lehmkühler

Supporting Information : Microsecond hydrodynamic interactions in dense colloidal dispersions probed at the European XFEL

Francesco Dallari,[a*] Avni Jain,[a] Marcin Sikorski,[b] Johannes Möller,[b] Richard Bean,[b] Ulrike Boesenberg,[b] Lara Frenzel,[a] Claudia Goy,[a] Jörg Hallmann,[b] Yoonhee Kim,[b] Irina Lokteva,[a,c] Verena Markmann,[a] Grant Mills,[b] Angel Rodriguez-Fernandez,[b] Wojciech Roseker,[a] Markus Scholz,[b] Roman Shayduk,[b] Patrik Vagovic,[b] Michael Walther,[a] Fabian Westermeier,[a] Anders Madsen,[b] Adrian P. Mancuso,[b,d] Gerhard Grübel[a,c] and Felix Lehmkühler[a,c]

[a]*Deutsches Elektronen-Synchrotron (DESY), Notkestraße 85, 22607 Hamburg, Germany.*, [b]*European X-ray Free-Electron Laser, 22869 Schenefeld, Germany,* [c]*The Hamburg Centre for Ultrafast Imaging, 22761 Hamburg, Germany, and* [d]*Department of Chemistry and Physics, La Trobe Institute for Molecular Science, La Trobe University, Melbourne, VC 3086, Australia. E-mail: francesco.dallari@desy.de*

1. Data elaboration

In order to match the repetition rate of the pulses within a single train of the European XFEL, AGIPD is built in such a way that the recorded MHz signals are directly saved in the pixels, each having 352 memory cells. The peculiarity is that these memory cells are physically distinct and separate, thus each of them had different offsets, noise levels and gain requiring a specific data calibration. It is known that each ASICS chip

is affected by a slightly different baseline value even after the background subtraction. To compensate this effect, we identify the ASICS that received a low number of photons and we correct this baseline value subtracting the median of the ASICS. For chips which have recorded stronger signals, the median value of the closest low intensity ASICS is used. After that, the speckle patterns expressed as a number of photons are obtained by normalizing the patterns by the ADU value of a single photon event (determined from the histogram of the data). In the present experiment all the measurement were performed keeping an overall low intensity, hence all the data are in high gain mode of AGIPD.

2. Expression for the experimental form factor

A precise evaluation of the measured scattered intensity has to consider the blurring due to finite physical size of the pixels. The signal from each pixel with surface σ will be the integral of the intensity over the all the q seen by σ, the expression used for the experimental form factor evaluation is then

$$P_{\mathrm{exp}}(q) = \int_\sigma dq' P_m(q) = \int_\sigma dq' \frac{\int_0^\infty dR p(R) f_{\mathrm{sph}}^2(q'R) V_{\mathrm{sph}}^2}{\int_0^\infty dR p(R) V_{\mathrm{sph}}^2}, \tag{1}$$

where $f_{\mathrm{sph}}(qR) = 3\left[\sin(qR) - qR\cos(qR))\right]/(qR)^3$ and $V_{\mathrm{sph}} = (4/3)\pi R^3$ are the spherical amplitude and volume respectively. The size distribution is modeled with the Schulz-Zimm function,

$$p(R) = \frac{R^z}{\Gamma(z+1)} \left(\frac{z+1}{R_0}\right)^{z+1} \exp\left(-\frac{z+1}{R_0} R\right) \tag{2}$$

where $\Gamma()$ is the Gamma function, R_0 is the mean radius and z is given by the size dispersity via $z = (\Delta R/R_0)^{-2} - 1$.

2.1. Decoupling amplitude factor

In the following the decoupling amplitude factor $X(q)$ is briefly introduced, for a more complete description, please refer to e.g. (Nägele, 1996; Westermeier *et al.*, 2012). When the charge dispersity is small enough to be neglected in first approximation, it is possible to attribute all the dispersity effects to the distribution of scattering amplitudes. Under these conditions, it is possible to express the measured structure factor ($S_m(q)$) as :

$$S_m(q) = [1 - X(q)] + X(q)S(q), \tag{3}$$

where $S(q)$ is the ideal structure factor of the monodisperse suspension and

$$X(q) = \frac{\langle f_{sph} \rangle^2}{\langle f_{sph}^2 \rangle} = \frac{[\int_0^\infty dRp(R) f_{sph}(q'R) V_{sph}]}{P_m(q) \int_0^\infty dRp(R) V_{sph}^2}. \tag{4}$$

This decoupling amplitude starts from the limit $X(q \to 0) \approx 1 - 9(\Delta R/R_0)^{-2}$, while in the large q limit $X(q \to \infty) \to 0$. Between these two limits displays an oscillatory behaviour with the largest amplitude shortly before the first minimum of $P_m(q)$.

3. Models for heated samples

This paragraph is a short summary of the models described in the supplementary information of (Lehmkühler *et al.*, 2020). When the X-ray pulse hits the sample we can consider the nanoparticles and the solvent as two separate entities. The water is subjected to an initial temperature jump of

$$\Delta T_{H2O} = \frac{E_{pulse}/\sigma^2}{2\pi c_p \rho_{H2O} \lambda_{abs}/(4\ln(2))}$$

where ρ_{H2O} is the mass density of water, λ_{abs} is the absorption length, c_p is the heat capacity and σ^2 is the beam size. Hence, we obtain that the temperature jump is proportional to the fluence $\Delta T_{H2O} \propto E_{pulse}/\sigma^2$. The heated volume try to reach the surrounding temperature of the unexcited sample with a characteristic time τ_0 given

by,

$$\tau_0 = \frac{c_p \rho \sigma^2}{2K_W},$$

where K_W is the thermal conductivity. With beams of 3.6 μm$\times$ 4.4 μm we have $\tau_0 \sim 55\mu$s, which is much longer than the time interval between two pulses even at the lowest repetition rates employed in the present work, furthermore, since in the MID experiment we worked with larger beams of 8 μm$\times$ 8 μm and 10μ m$\times$ 10 μm, in that case $\tau_0 \approx$223 μs and 348 μs respectively. This means also that in XPCS experiments with μs resolution, the scattering volume will never remain at the initial temperature.

After N pulses with a time separation δt the temperature of the bulk water will be described by:

$$T_n = \Delta T_{H_2O} \sum_{n=0}^{N} \frac{1}{1 + (n\delta t/\tau_0)} + T_0$$

where T_0 is the initial temperature. It is important to notice that the limit of $T_n \approx N\Delta T_{H_2O}$ will be reached only in situations where $\tau_0 \gg N\delta t$, which was never encountered in the present work because even for largest beams we had only $\tau_0 \sim 2(N\delta t)$.

From the point of view of the nanoparticles, the temperature jump is described with:

$$\Delta T_P = \frac{\sigma_P^2 E_{pulse}/\sigma^2}{\rho_P c_p^P V_P}$$

where ρ_P, c_P and V_P are the density, heat capacity and volume of the nanoparticles, while $\sigma_P^2 = V_P/\lambda_P$ is the cross-section. The small size of the nanoparticles allows them to maintain the same temperature within their volume even in the cooling process. The time evolution of the temperature of a given particle will follow then an exponential law with a characteristic time given by :

$$\tau_P = \left(3\frac{K}{K_w}Bi\frac{\alpha}{R^2}\right)^{-1}$$

where K is the nanoparticle thermal conductivity, $\alpha = K/(\rho_P c_p^P)$ and $Bi = hR/K$, known also as Biot number. The parameter h is the convective heat transfer coefficient from water, whose value can span over 2 decades. Smaller particles will require less time to relax at the solvent temperature, and for sizes of tens of nanometers like in the present work, $\tau_P \sim 62$ ns.

4. I(q), S(q) and H(q) comparison

In figure 1 we report the scattered intensity together with the fitted structure factor and hydrodynamic functions for samples A ($\phi = 0.2$), B ($\phi = 0.1$) and C ($\phi = 0.05$). It is possible to see how the distance between H(q) and S(q) increases with concentration indicating the presence of stronger effects of the HI on the dynamics in more crowded environments.

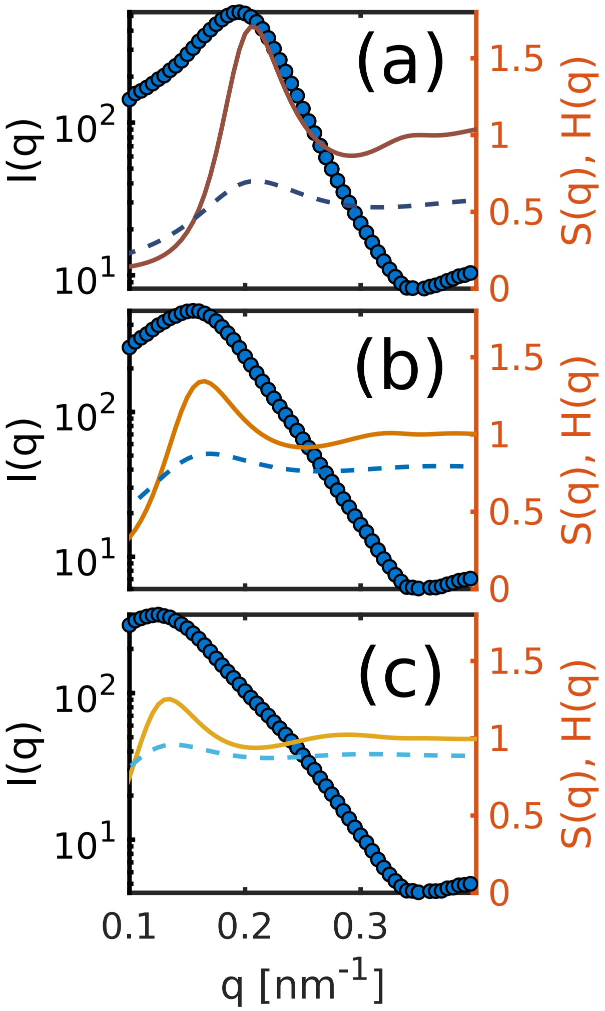

Fig. 1. Here we report the I(q) (filled circles) together with the fitted S(q) (continuous lines) and the H(q) (dashed lines) for samples A (a), B (b) and C (c).

5. Sample damage

During the experiment we probed even higher fluences than the ones reported in the main text. For fluences higher than 16 mJ/mm^2 the dynamics becomes so fast that only few pulses stays correlated making a reliable dynamical investigation impossible. For this reason we skipped the higher fluences in the main text. However, it is still

possible to use the information derived from the I(q) to infer a damage threshold. In figure 2 we report the normalized I(q) across the train of pulses for various fluences. It is possible to notice how the I(q) remains stable across the train up to 27.7 mJ/mm^2, while for higher fluences the shape of the I(q) evolves with the number of pulses. Notably the position of the minima in the I(q) does not change appreciably, meaning that the particles maintain their spherical shape, as observed also with larger particles in (Lehmkühler *et al.*, 2020). Macroscopically, this evolution is associated with the formation of bubbles in the capillary observed after the illumination. However, the characterization at very high fluences was outside the aims of the experiment and with our current knowledge we cannot be sure if this structural change is a precursor of the phase transition in the solvent or the effect of a strong charge destabilization operated by the X-rays. Nevertheless, we can safely assume that our time resolved model will not be valid for fluences above 27.7 mJ/mm^2.

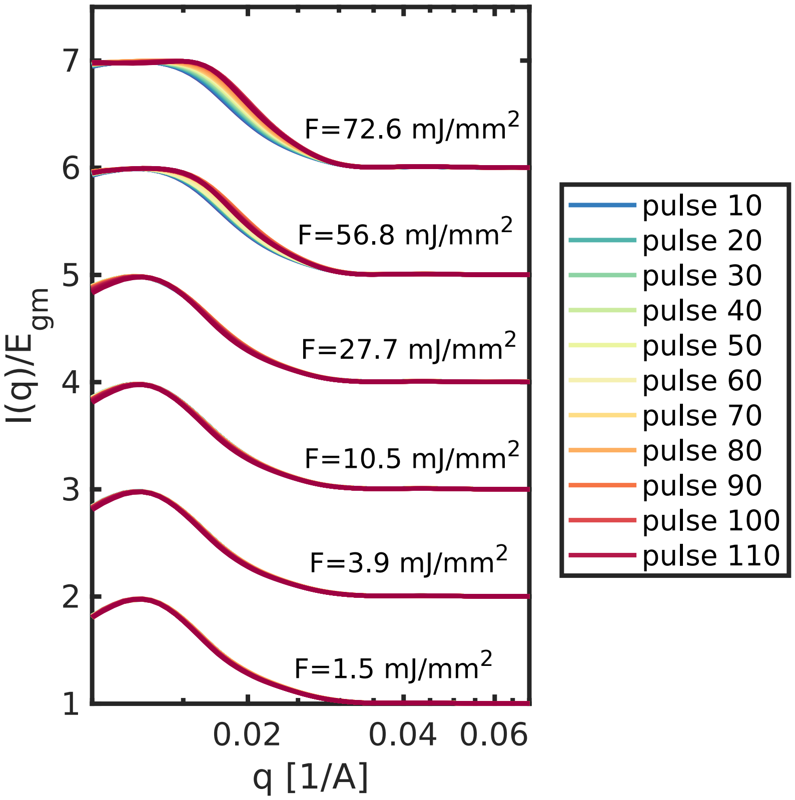

Fig. 2. Normalized $I(q, n_p)$ plotted for various fluences. The curves have been shifted and reported in linear scale to highlight the modifications developing in the structure for higher fluences.

6. I(q) from MID samples

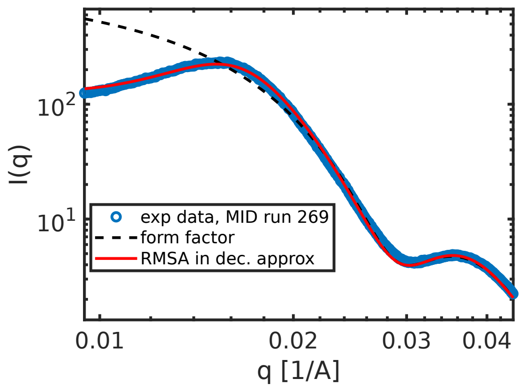

Fig. 3. Example of the I(q) obtained in a run of the MID experiment. The data points are still fitted by the same function described in the main text, with the only differences given by the values of the radius (R=15 nm) and volume fraction ($\phi = 0.22$)

7. Table of runs and parameters

SPB/SFX runs. Here the absorbers are made of stacks of silicon slabs.

run number	vol. frac.	abs. thickness [μm]	fluence [mJ/mm^2]	num. pulses	rep.rate [MHz]
44	0.20	1200	0.2	120	1.128
45	0.20	1200	0.2	120	1.128
46	0.20	1000	1.5	120	1.128
47	0.20	1000	1.5	120	1.128
48	0.20	900	3.9	120	1.128
49	0.20	900	3.9	120	1.128
50	0.20	800	10.4	120	1.128
51	0.20	800	10.5	120	1.128
144	0.10	1000	1.5	120	1.128
145	0.10	1000	1.5	120	1.128
146	0.10	1000	1.5	120	1.128
147	0.10	1000	1.5	120	1.128
148	0.10	900	3.9	120	1.128
149	0.10	900	4.0	120	1.128
150	0.10	800	10.5	120	1.128
151	0.10	800	10.4	120	1.128
152	0.10	700	28.1	120	1.128

153	0.10	700	28.3	120	1.128
164	0.05	1000	1.4	120	1.128
165	0.05	1000	1.5	120	1.128
166	0.05	1000	1.5	120	1.128
167	0.05	1000	1.4	120	1.128
168	0.05	900	3.9	120	1.128
169	0.05	900	3.9	120	1.128
170	0.05	800	10.3	120	1.128
171	0.05	800	10.3	120	1.128
172	0.05	700	27.3	120	1.128
173	0.05	700	27.2	120	1.128
174	0.05	625	59.0	120	1.128
175	0.05	625	59.2	120	1.128
176	0.05	600	76.1	120	1.128
250	0.10	1200	0.1	120	1.128
251	0.10	1200	0.1	120	1.128
252	0.10	1200	0.1	120	1.128
253	0.10	1200	0.1	120	1.128
305	0.10	1000	0.6	60	0.564
306	0.10	1000	0.5	60	0.564
307	0.10	800	4.0	60	0.564
308	0.10	800	3.9	60	0.564
313	0.10	1000	0.3	40	0.376
314	0.10	1000	0.3	40	0.376
315	0.10	800	2.1	40	0.376
316	0.10	800	2.2	40	0.376

MID runs. Here the absorbers are made of stacks of CVD diamond windows.

run number	vol. frac.	abs. thickness [μm]	fluence [mJ/mm^2]	num. pulses	rep.rate [MHz]
137	0.22	4500	2.5	199	2.257
138	0.22	4100	3.8	199	2.257
139	0.22	3700	6.0	199	2.257
140	0.22	3300	9.0	199	2.257
141	0.22	2900	14.0	199	2.257
269	0.22	4500	2.5	175	1.128
270	0.22	4100	3.9	175	1.128
271	0.22	3700	5.8	175	1.128
272	0.22	3300	9.1	175	1.128
273	0.22	2900	13.2	175	1.128
274	0.22	2500	20.9	175	1.128
329	0.22	4500	2.7	86	0.564
330	0.22	4100	4.1	86	0.564
331	0.22	3700	6.4	86	0.564
332	0.22	3300	10.3	86	0.564
333	0.22	2900	14.8	86	0.564
334	0.22	2500	23.0	86	0.564
514	0.22	4725	1.6	143	1.128
515	0.22	4325	2.3	143	1.128
516	0.22	3925	3.6	143	1.128
517	0.22	3125	8.3	143	1.128
518	0.22	2725	12.7	143	1.128
519	0.22	2525	16.0	143	1.128

References

Lehmkühler, F., Dallari, F., Jain, A., Sikorski, M., Möller, J., Frenzel, L., Lokteva, I., Mills, G., Walther, M., Sinn, H., Schulz, F., Dartsch, M., Markmann, V., Bean, R., Kim, Y., Vagovic, P., Madsen, A., Mancuso, A. P. & Grübel, G. (2020). *Proceedings of the National Academy of Sciences*, **117**(39), 24110–24116.
URL: *https://www.pnas.org/content/117/39/24110*

Nägele, G. (1996). *Physics Reports*, **272**(5), 215 – 372.
URL: *http://www.sciencedirect.com/science/article/pii/037015739500078X*

Westermeier, F., Fischer, B., Roseker, W., Grübel, G., Nägele, G. & Heinen, M. (2012). *The Journal of Chemical Physics*, **137**(11), 114504.
URL: *https://doi.org/10.1063/1.4751544*
